# Supplementary material for: Constraining tectonic uplift and advection from the main drainage divide of a mountain belt
Source: Nat Commun. 2021 Jan 22;12:544. doi: 10.1038/s41467-020-20748-2 (PMC7822862; doi:10.1038/s41467-020-20748-2)
Supplement: Supplementary file 1 — Supplementary Information [file 41467_2020_20748_MOESM1_ESM.pdf]

Supplementary Information for

# Constraining tectonic uplift and advection from the main drainage divide of a mountain belt

Chuanqi He[1,2], Ci-Jian Yang[2], Jens M. Turowski[2], Gang Rao[1 *],

Duna C. Roda-Boluda[2], Xiao-Ping Yuan[2]

[1] Key Laboratory of Geoscience Big Data and Deep Resource of Zhejiang Province, School of Earth Sciences, Zhejiang University, Hangzhou 310027, China

[2] GFZ German Research Centre for Geosciences, Potsdam 14473, Germany

*Corresponding author: Gang Rao (raogang@zju.edu.cn)

## Contents of this File

Supplementary Figs. 1–13;

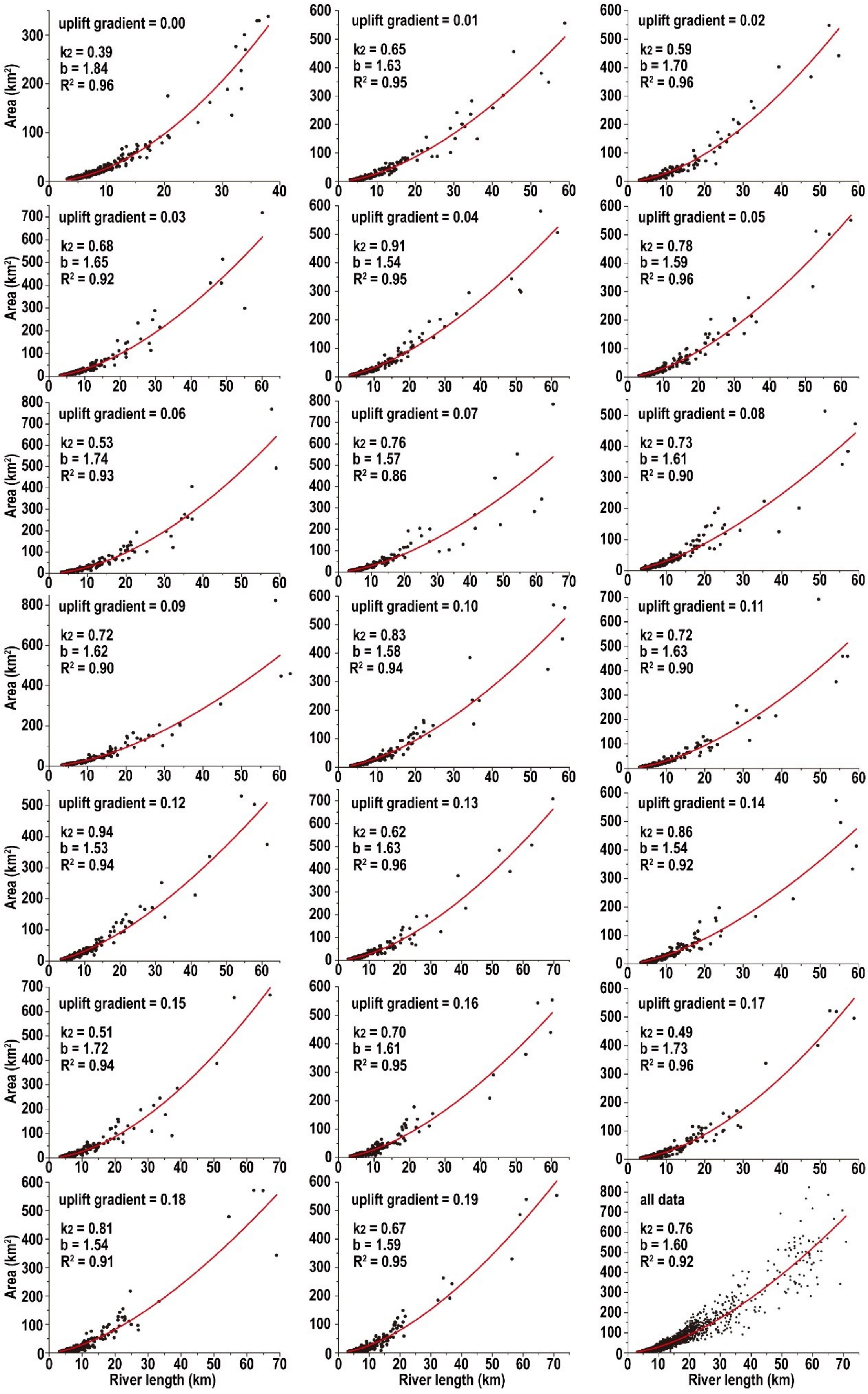


**Supplementary Fig. 1 Hack's parameters derived from 20 numerical models with**

**different uplift gradients.** $k_2$ and $b$ are Hack's parameters (see Methods). The topographies of 20 numerical models at 300 Myr (million years) are analysed. All drainage basins with areas of more than 5 $km^2$ are extracted for analysis. Source data are provided as a Source Data file.

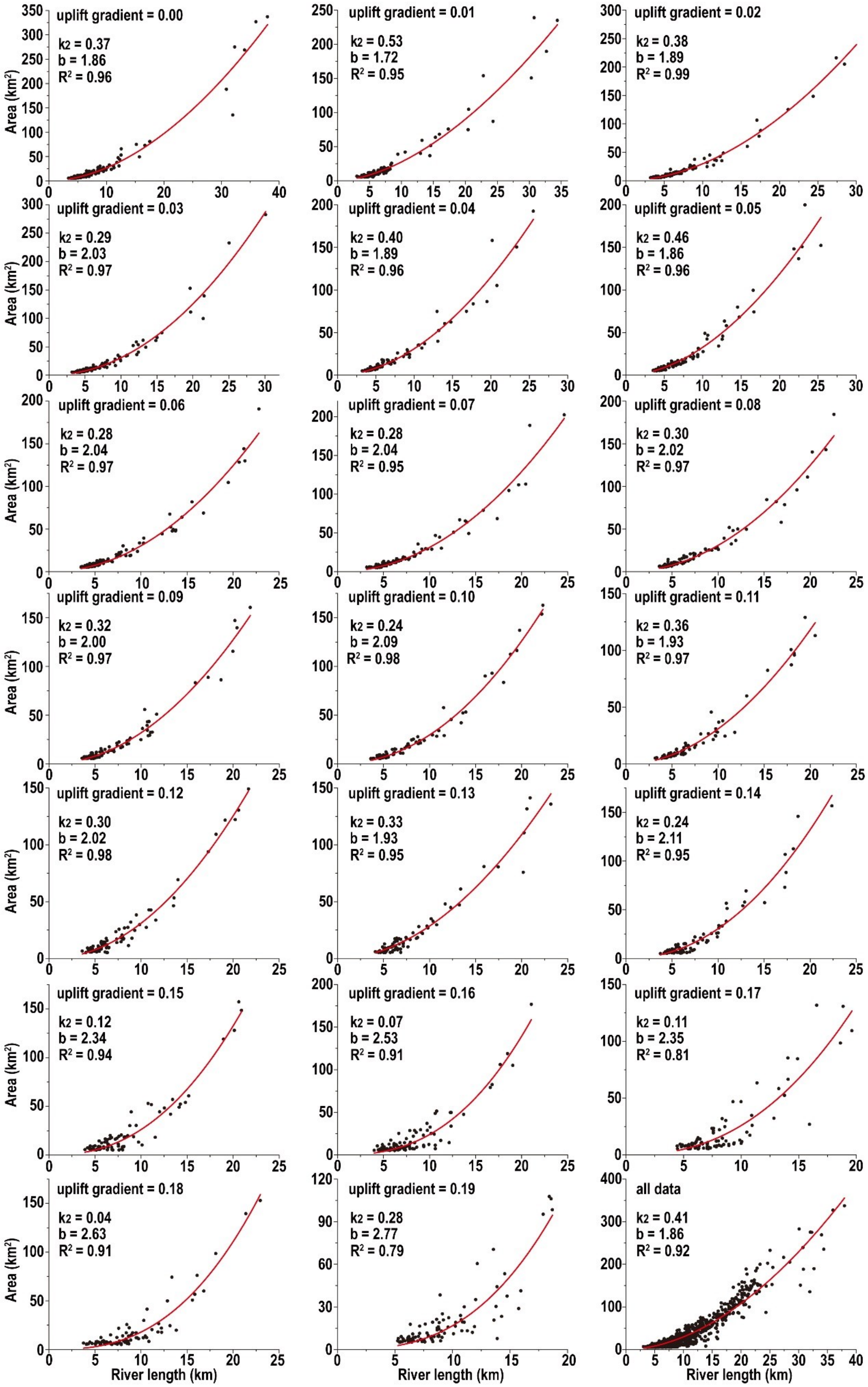


**Supplementary Fig. 2 Hack's parameters derived from drainage basins on the top side**

**of the 20 numerical models.** The topographies of 20 numerical models at 300 Myr are analysed. All drainage basins with areas of more than 5 $km^2$ are extracted for analysis. Source data are provided as a Source Data file.

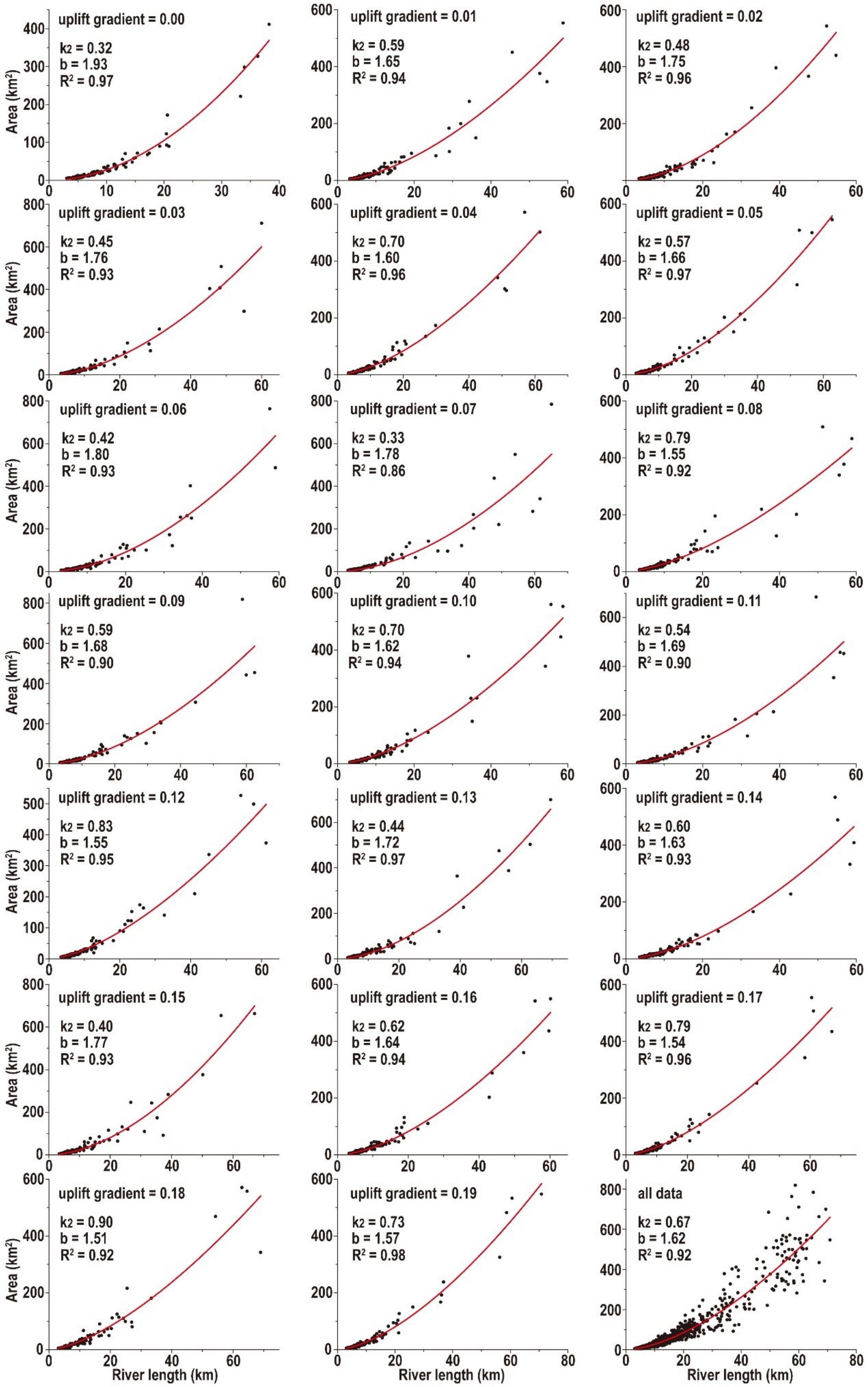


**Supplementary Fig. 3 Hack's parameters derived from drainage basins on the bottom**

**side of the 20 numerical models.** The topographies of 20 numerical models at 300 Myr are analysed. All drainage basins with areas of more than 5 km$^2$ are extracted for analysis. Source data are provided as a Source Data file.

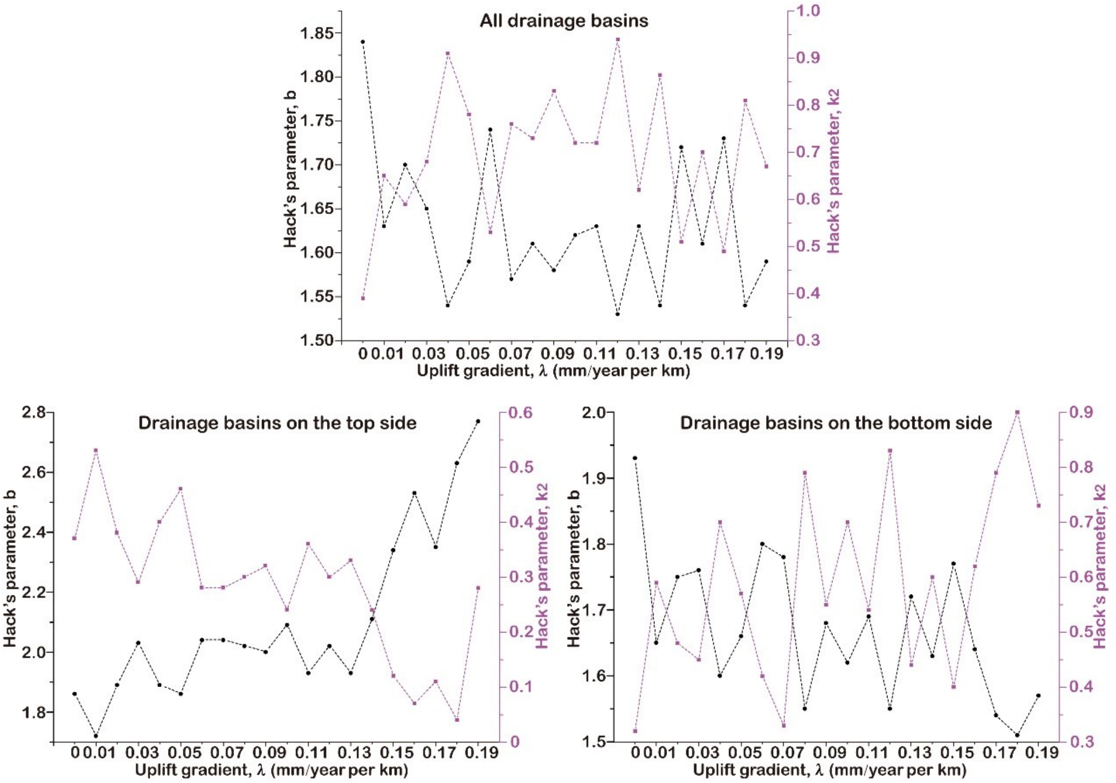


**Supplementary Fig. 4 Hack's parameters vary with uplift gradient.** The river length and drainage area data can be found in Supplementary Figs. 1–3. Source data are provided as a Source Data file.

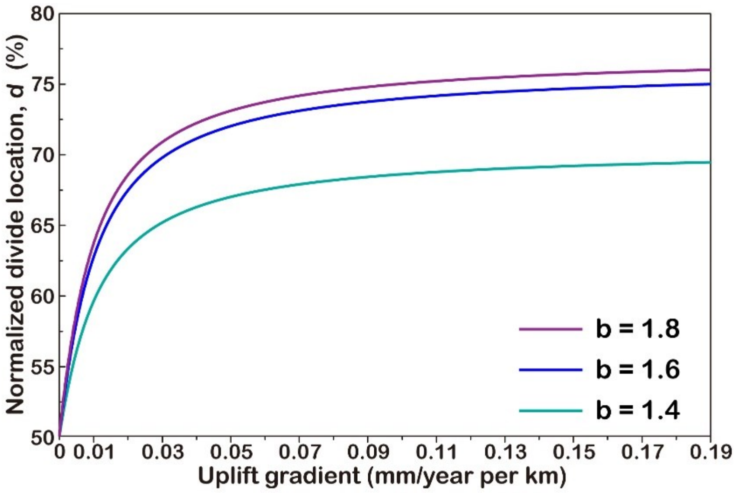


**Supplementary Fig. 5 Hack's parameter (*b*) influences divide location for models with different uplift gradients.** These plots are based on equation (9) (see Methods).

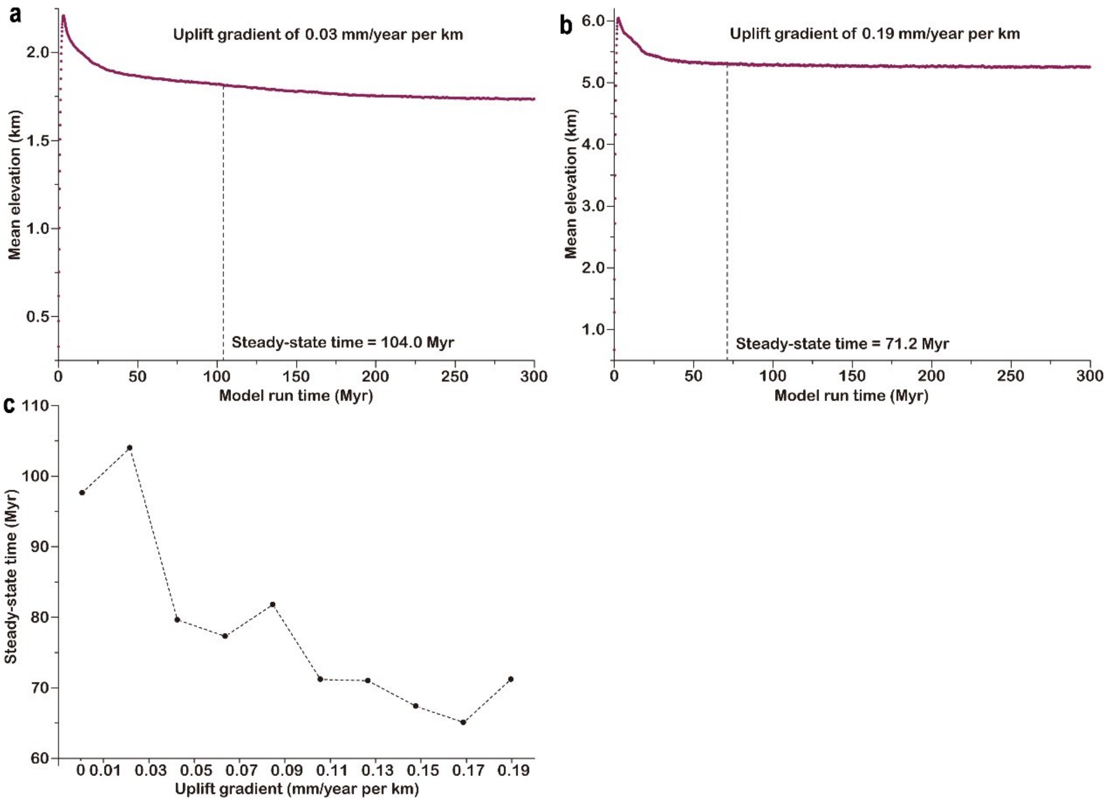


**Supplementary Fig. 6 Time needed to reach steady state. a**, **b**, The mean elevation with model run time showing steady-state time. **c**, The steady-state time varies with uplift gradient. Source data are provided as a Source Data file.

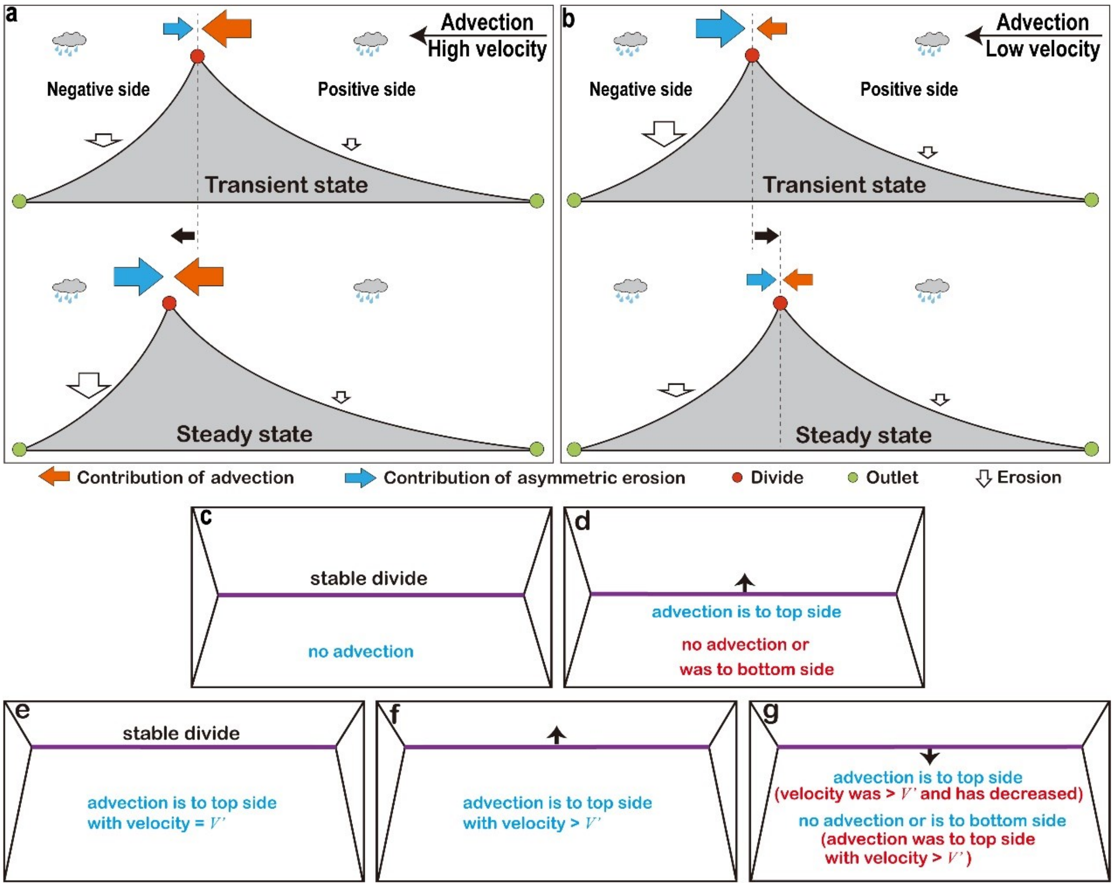


**Supplementary Fig. 7 Divide migration in response to advection and erosion, and its implications for deriving tectonic information. a**, When advection dominates divide mobility, divide moves towards the negative side until it reaches a steady state. **b**, When erosional contrast dominates divide mobility, divide migrates towards the positive side to reach steady state. **c**, Symmetric mountain belt with a stable divide. **d**, Symmetric mountain belt with an unstable divide. **e**, Asymmetric mountain belt with a stable divide. **f**, Asymmetric mountain belt with the divide migrating towards the steeper side. **g**, Asymmetric mountain belt with the divide moving towards the gentler-sloping side. Purple lines are the divide, with its direction of motion marked by arrows. Using the theoretical relationship (Fig. 3a), we can estimate an advection velocity *V*' from the current divide location. Constraints on the present advection and its history are given in the blue and red comments, respectively.

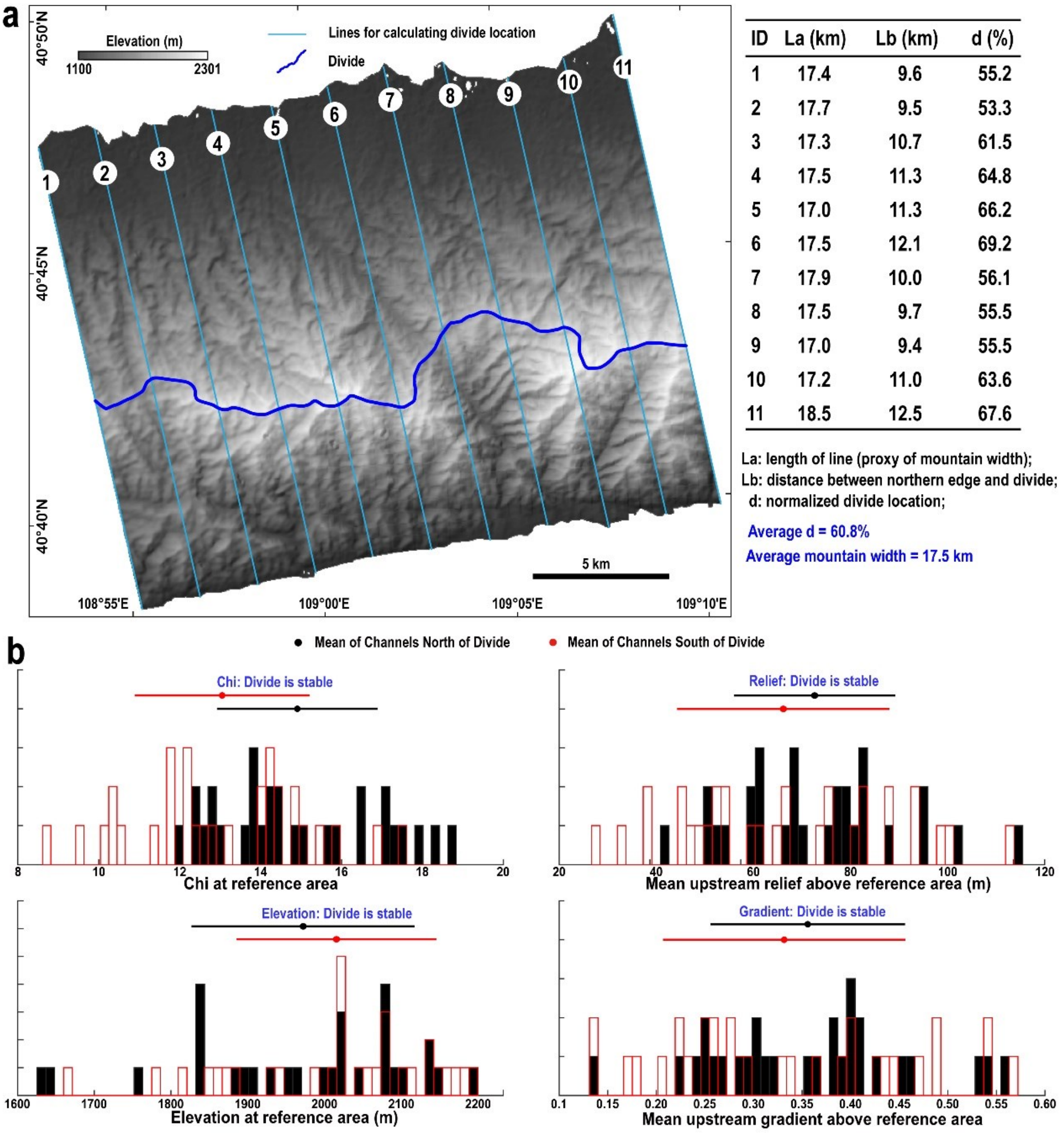


| ID | La (km) | Lb (km) | d (%) |
|---|---|---|---|
| 1 | 17.4 | 9.6 | 55.2 |
| 2 | 17.7 | 9.5 | 53.3 |
| 3 | 17.3 | 10.7 | 61.5 |
| 4 | 17.5 | 11.3 | 64.8 |
| 5 | 17.0 | 11.3 | 66.2 |
| 6 | 17.5 | 12.1 | 69.2 |
| 7 | 17.9 | 10.0 | 56.1 |
| 8 | 17.5 | 9.7 | 55.5 |
| 9 | 17.0 | 9.4 | 55.5 |
| 10 | 17.2 | 11.0 | 63.6 |
| 11 | 18.5 | 12.5 | 67.6 |

**Supplementary Fig. 8 Location and mobility of the main drainage divide of Wula Shan horst (Northern China). a***,* The whole 20-km-wide natural landscape is divided into 10 parts with 11 lines that are perpendicular to the trend of the mountain belt. **b**, Ref.[1] developed a tool (DivideTools) to evaluate the stability of divide using topographic metrics (Chi, elevation, relief, and gradient) near the divide. In this study, the divide stability in Wula Shan horst is assessed using this tool. The main drainage divide is predicted to be stable, which implies that these landscapes are at steady state. The reference area over which the divide values are computed is set to be 0.01 km$^2$.

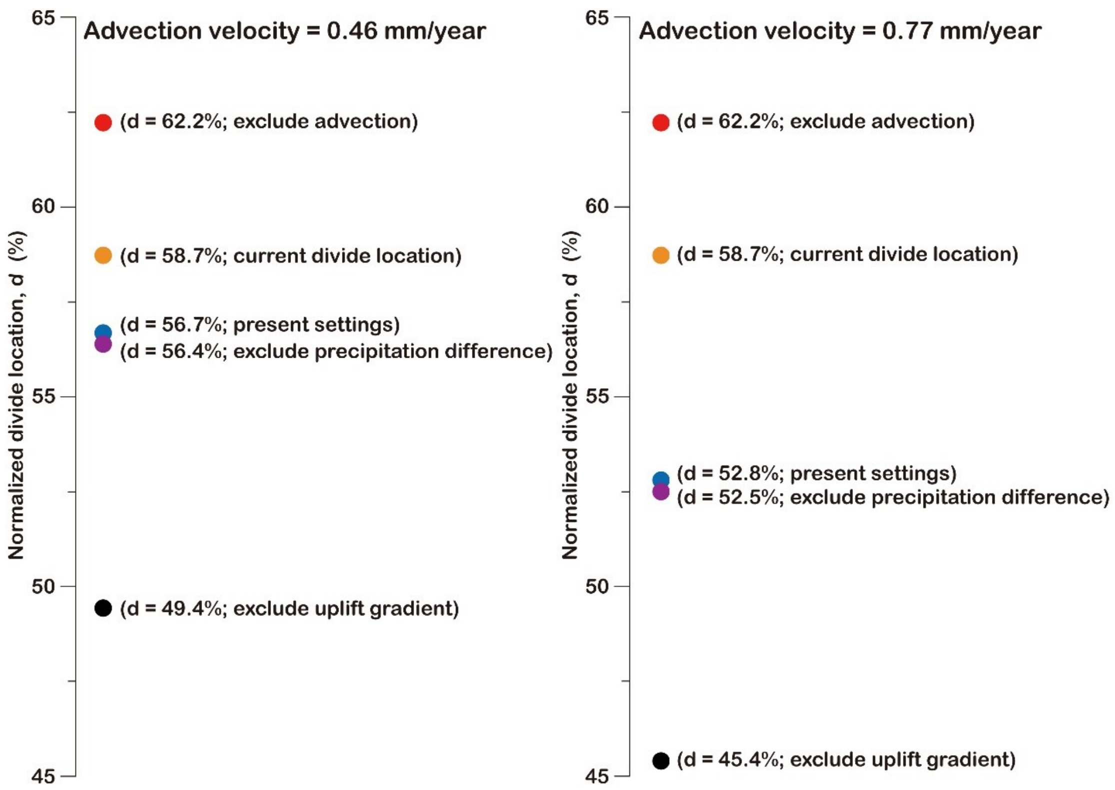


**Supplementary Fig. 9 Divide locations of Northeastern Sicily under different settings.**

The divide locations are calculated using the MATLAB script developed by this work (divide_location.m, see Code availability). The present settings for Northeastern Sicily are as follows: the uplift rate linearly increases from 0.85 mm/year at the edge of the negative side (NW edge) to 1.22 mm/year at the edge of the positive side (SE edge), with an uplift gradient of 0.0092 mm/year per km; the advection velocities are 0.46 mm/year and 0.77 mm/year for the left and right figures, respectively; the precipitation rates of the NW and SE sides are 711 mm/year and 705 mm/year, respectively; other values including Hack's parameters, hillslope length, and erodibility can be found in the Methods section. If we exclude the precipitation difference, a uniform precipitation rate of 700 mm/year is used. If we exclude the uplift gradient, a uniform uplift rate of 1 mm/year is used.

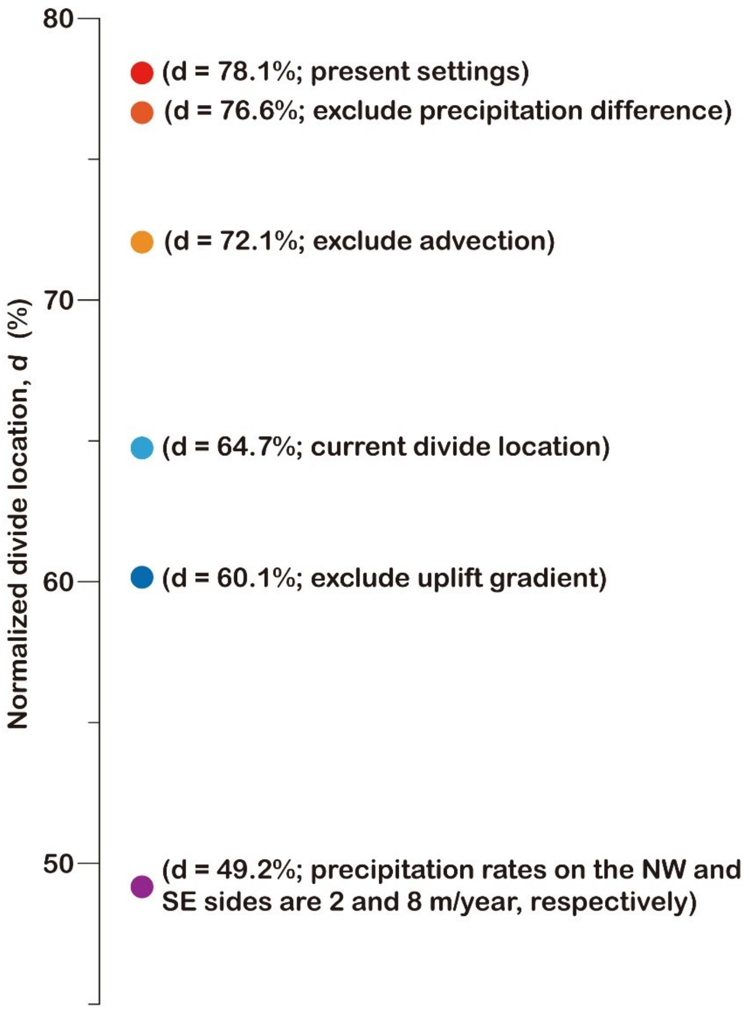


**Supplementary Fig. 10 Divide locations of Southern Taiwan under different settings.** The divide locations are calculated using the MATLAB script developed by this work (divide_location.m, see Code availability). The present settings for Southern Taiwan are as follows: the uplift rate linearly increases from 0.5 mm/year at the edge of the positive side to 18.7 mm/year at the edge of the negative side, with an uplift gradient of 0.14 mm/year per km; the advection velocity linearly increases from 0 mm/year at the edge of the negative side to 45.5 mm/year at the edge of the positive side, with an advection velocity gradient of 0.35 mm/year per km; the precipitation rates of the NW and SE sides are 2528 mm/year and 2231 mm/year, respectively; other values including Hack's parameters, hillslope length, and erodibility can be found in the Methods section. If we exclude the precipitation difference, a

uniform precipitation rate of 2000 mm/year is used. If we exclude the uplift gradient, a uniform uplift rate of 9.6 mm/year is used.

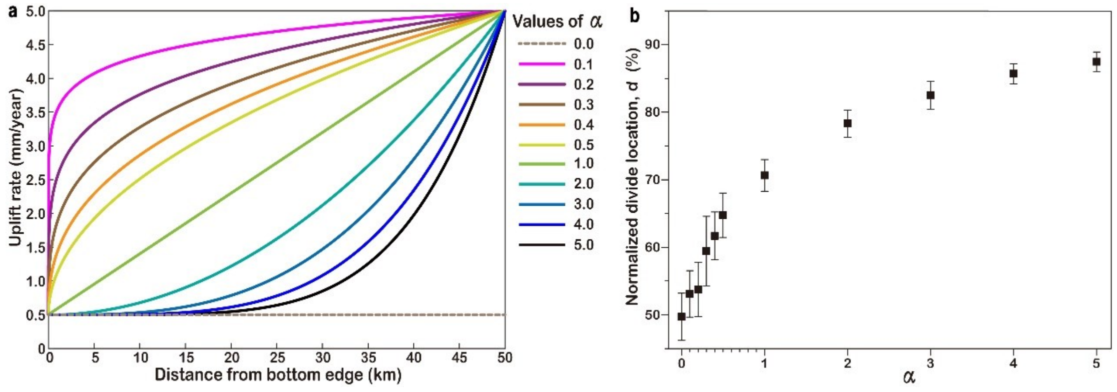


**Supplementary Fig. 11 Sensitivity of the exponent (*α*) determining the pattern of uplift gradient in influencing divide location. a**, The uplift pattern with different *α*. **b**, The relationship between *d* and *α*. We assume that the uplift rate at the bottom edge is 0.5 mm/year, which increases to 5.0 mm/year at the top edge (except the uniform uplift case, where the uplift rate is 0.5 mm/year). The uplift rate across the model domain is described by a power law as

$$U = U_0 + cL^{\alpha} ,$$

where $U$ is the uplift rate at a point with a distance of $L$ to the bottom edge. $U_0$, with a value of 0.5 mm/year, is the uplift rate at the bottom edge. The exponent of the power law $\alpha$ varies from 0 to 5. When $\alpha = 0$, the uplift rate is uniform (0.5 mm/year); when $\alpha = 1$, the uplift rate linearly increases from the bottom edge of 0.5 mm/year to 5.0 mm/year at the top edge; for other $\alpha$ values, the uplift rate increases nonlinearly from the bottom edge of 0.5 mm/year to 5.0 mm/year at the top edge. The coefficient $c$ can be calculated based on the uplift rate at two edges and the value of $\alpha$. Source data are provided as a Source Data file.

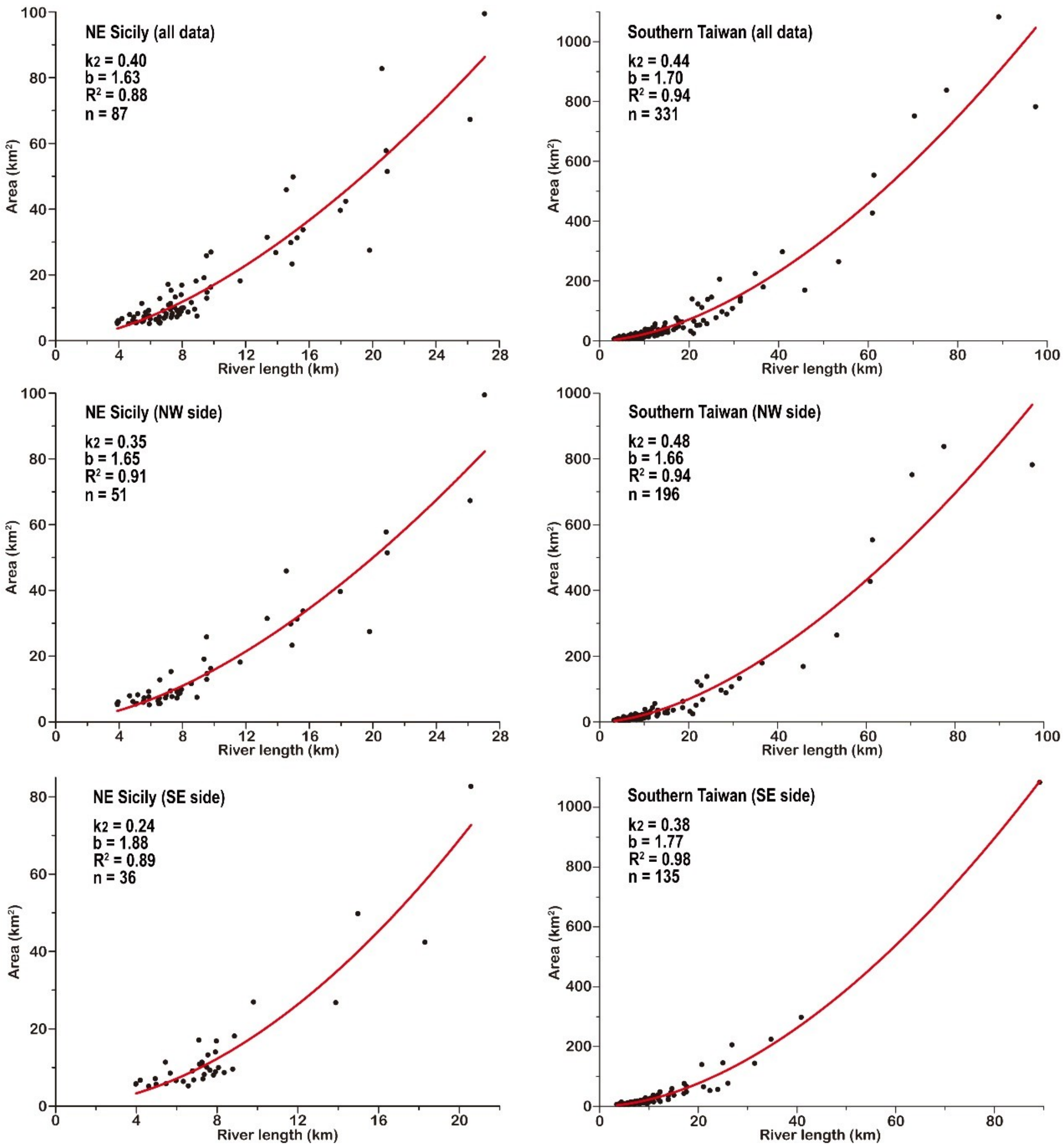


**Supplementary Fig. 12 Hack's parameters for Northeastern Sicily and Southern Taiwan.** The drainage area threshold for the analysis is 5 km$^2$. Source data are provided as a Source Data file.

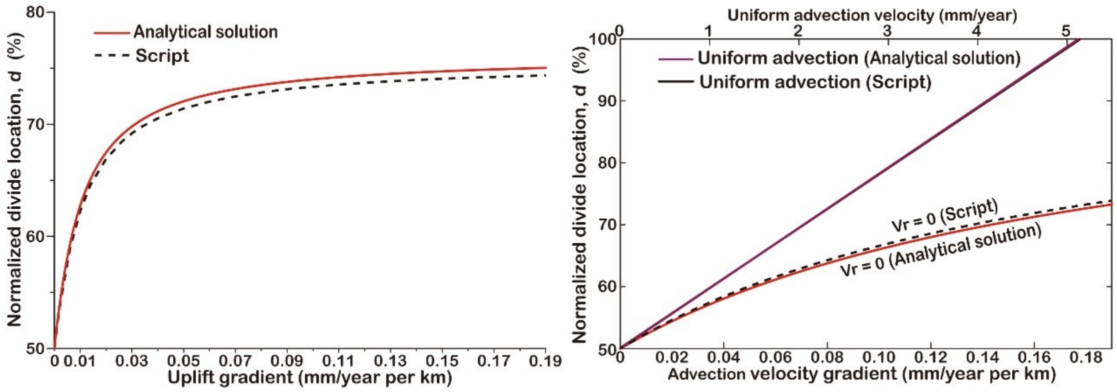


**Supplementary Fig. 13 Comparison of divide locations calculated by the analytical solutions and the MATLAB script (divide_location.m).** The analytical solutions are independent of Hack's coefficient, $k_2$, while the script requires $k_2$. The results show that the analytical solutions and the script match best with a $k_2$ value of 1. For uplift gradient, the result of the analytical solution is slightly higher than that of the script. For uniform advection, the analytical solution and the script have the same result. For constant shortening, the result of the analytical solution is slightly lower than that of the script.

## Supplementary References

1. Forte, A. M., & Whipple, K. X. Criteria and tools for determining drainage divide stability. *Earth Planet. Sci. Lett.* **493**, 102–117 (2018).
