# Supplementary material for: Constraining tectonic uplift and advection from the main drainage divide of a mountain belt
Source: Nat Commun. 2021 Jan 22;12:544. doi: 10.1038/s41467-020-20748-2 (PMC7822862; doi:10.1038/s41467-020-20748-2)
Supplement: Supplementary file 3 — Description of Additional Supplementary Files [file 41467_2020_20748_MOESM3_ESM.pdf]

Description of Additional Supplementary Files

File Name: Supplementary Movie 1
Description: Numerical simulation showing the landscape evolution in response to asymmetric uplift.
